# Lipoxygenase and Its Relationship with Ethylene During Ripening of Genetically Modified Tomato (*Solanum lycopersicum*)

**DOI:** 10.17113/ftb.58.02.20.6207

**Published:** 2020-06

**Authors:** Arturo Alberto Velázquez-López, Javier De La Cruz-Medina, Hugo Sergio García, Gilber Vela-Gutiérrez, Cristóbal Torres-Palacios, Elizabeth León-García

**Affiliations:** 1 Mexican National Technology/Technological Institute of Veracruz, 2779 M.A. de Quevedo, 91897 Veracruz, Mexico; 2 Faculty of Food and Nutrition Sciences, University of Science and Arts of Chiapas, 1150 Northwest bypass, 29000 Tuxtla Gutiérrez, Chiapas, Mexico; 3 National Institute of Forestry, Agricultural and Livestock Research, La Posta Experimental Field, Km 22.5 Federal Highway Veracruz-Córdoba, Medellín de Bravo, 94277 Veracruz, Mexico

**Keywords:** lipoxygenase activity, *TomloxB*, ethylene production, 1-aminocyclopropane-1-carboxylic acid oxidase, tomato ripening

## Abstract

**Research background:**

TomloxB is the main isoform of lipoxygenase associated with ripening and senescence of fruits. On the other hand, ethylene, a gaseous hormone, is essential for the regulation of ripening in climacteric fruits like tomatoes. However, the relationship between TomloxB and ethylene production has not been thoroughly studied. Therefore, we aim to assess the effect of exogenous ethylene in transgenic tomatoes that contain a silenced *TomloxB* gene, and subsequently evaluate lipoxygenase activity, 1-aminocyclopropane-1-carboxylic acid oxidase and ethylene production; as well as to quantify the expression of the genes encoding 1-aminocyclopropane-1-carboxylic acid oxidase and *TomloxB.*

**Experimental approach:**

To investigate the effect of lipoxygenase and 1-aminocyclopropane-1-carboxylic acid oxidase activity, fruits harvested at the stages of break, turning and pink were used. Tomatoes at break stage collected from transgenic and wild type plants were used to determine ethylene production and gene expression. Genetically modified and wild type tomato fruits were exposed to 100 μL/L exogenous ethylene. Lipoxygenase activity was measured spectrophotometrically. Activity of 1-aminocyclopropane-1-carboxylic acid oxidase and ethylene production were determined by gas chromatography. Oligonucleotides for differentially expressed genes: 1-aminocyclopropane-1-carboxylic acid oxidase and *TomloxB* were used to determine gene expression by real-time PCR.

**Results and conclusions:**

The data showed that silencing of *TomloxB* caused a reduction in lipoxygenase activity and ethylene production in tomato fruits, and also reduced 1-aminocyclopropane-1-carboxylic acid oxidase activity. Hence, the addition of exogenous ethylene increased lipoxygenase activity in all treatments and 1-aminocyclopropane-1-carboxylic acid oxidase activity only in transgenic lines at break stage, consequently there was a positive regulation between *TomloxB* and ethylene, as increasing the amount of ethylene increased the activity of lipoxygenase. The results suggest that lipoxygenase may be a regulator of 1-aminocyclopropane-1-carboxylic acid oxidase and production of ethylene at break stage.

**Novelty and scientific contribution:**

These results lead to a better understanding of the metabolic contribution of TomloxB in fruit ripening and how it is linked to the senescence-related process, which can lead to a longer shelf life of fruits. Understanding this relationship between lipoxygenase and ethylene can be useful for better post-harvest handling of tomatoes.

## INTRODUCTION

Tomato maturation involves gene expression and complex biochemical changes, which result in the alteration of physiological properties leading to changes in colour, texture, taste and aroma (*1*, *2*). Senescence and ripening of fruits are characterized by an early deterioration of their cellular membranes (*3*). Lipoxygenases (LOX, EC 1.13.11.12), widely distributed in plants, were first purified in 1947 (*4*). These include a class of iron-dependent enzymes that use molecular oxygen for the dioxygenation of fatty acids containing a 1,4-pentadiene structure, such as linoleic and linolenic acids, which are two of the main fatty acids located in the plant cell membrane (*4*-*6*). However, the precise physiological and biochemical functions of plant lipoxygenases during fruit maturation are uncertain (*7*, *8*). LOX is associated with flavour and aroma formation (*5*, *9*), plant cell senescence, as a response to pest attack and wounds (defense) (*6*, *10*, *11*), and fruit ripening (*7*, *12*). On the other hand, 1-aminocyclopropane-1-carboxylic acid (ACC) oxidase (ACCO) is a member of a large Fe(II)-requiring dioxygenase/oxidase superfamily. In addition, ACCO is directly related to the production of ethylene from methionine and is the key regulatory enzyme (*13*). Furthermore, ethylene is a gaseous plant hormone that triggers the maturation process in climacteric fruits and influences the senescence and abscission of the plant organs (*14*). It has also been implicated in developmental processes such as seed germination, cell elongation, root formation, sex determination, pollination and flowering, and it regulates plant responses to biotic and abiotic stress (*14*, *15*), and is related to ripening (*16*, *17*), as well as the production of fruit metabolites that act as part of defense mechanisms. Griffiths *et al.* (*1*) determined that ethylene caused up-regulation of *TomloxB* expression in fruit but they did not study the effect of LOX on ethylene. Our hypothesis is that there is a relationship between LOX activity and ethylene or the limiting enzyme in ethylene production (ACCO). Hence, in the present study, the LOX and ACCO activity, ethylene production and gene expression levels (*TomloxB* and *ACCO*) of genetically modified tomato fruits (with reduced expression of the *TomloxB* gene) were measured and compared with fruits exposed to exogenous ethylene during maturation.

## MATERIALS AND METHODS

### Plant material

We used previously genetically transformed plant material prepared by León-García *et al.* (*17*). These tomato plants (*Solanum lycopersicum* var. TA234) contain tomato lipoxygenase (*TomloxB*) gene in anti-sense constructs (*17*). In this study three transgenic lines: A-TR, B-TR and C-TR were used; each transgenic line was evaluated with 20 fruits. Wild type and transgenic tomato plants were grown in a greenhouse under a 16/8 h photoperiod, with a temperature of (25±2) °C. Fruits were harvested in the stages of break, turning and pink. Lipoxygenase and ACCO activities were determined in fruits immediately after harvesting (*18*). Fruits were rinsed and frozen in liquid nitrogen and stored at -80 °C until further use.

### Ethylene production

The method suggested by De la Cruz *et al*. (*19*) for analysis of ethylene was slightly modified as follows: single fruits were placed for 1 h in individual 350-mL glass beakers, fitted with a septum. Then, the headspace was sampled by insertion of a 1-mL gas chromatography syringe and immediately injected into an Agilent CG 7820A gas chromatograph (Shanghai, PR China) fitted with a Poraplot Q column (15 m×0.32 mm, 20 μm), under the following conditions: injector at 150 °C; FID detector at 250 °C; initial oven at 90 °C, increased to 100 °C at 4 °C/min, held for 15 min, increased to 115 °C at 3 °C/min and held for 15 min. High-purity nitrogen was used as carrier gas at a flow rate of 1 mL/min. Ethylene was quantified by calculating concentrations using regression equations determined by injection of ten different concentrations of ethylene standard to obtain peak areas for calibration curve.

### Lipoxygenase activity

Tomato fruits were selected to determine the activity of lipoxygenase in the selected ripening stages. LOX activity was measured spectrophotometrically as described by Gökmen *et al.* (*20*) with modifications. Ten grams of fruit with removed seeds and placenta were weighed, chopped and homogenized in a porcelain mortar with pestle, and then 10 mL of 0.2 M phosphate buffer, pH=6.0 (J.T.Baker, Phillipsburg, NJ, USA) were added and centrifuged at 12 000×*g* (centrifuge model 5415 R; Eppendorf, Hamburg, Germany) at 10 °C.

The substrate was prepared by mixing 157.2 μL of linoleic acid (Sigma-Aldrich, Merck, St. Louis, MO, USA), 157.2 μL of Tween 80 and 10 mL of deionized water. This solution was filtered through a nitrocellulose filter (Millipore, Billerica, MA, USA). The solution was clarified by adding 1 mL of 1 M NaOH (Golden Bell, Chapultepec, Mexico City, Mexico) and diluted to 200 mL with 0.2 M phosphate buffer, pH=6.0 (J.T.Baker). A unit of LOX activity is defined as the increase in 0.001 absorbance at 234 nm per minute per mg of protein, measured using the Bradford method (*21*), and bovine serum albumin (Sigma-Aldrich, Merck) was used as standard.

### 1-Aminocyclopropane-1-carboxylic acid oxidase activity

Tomato fruits were selected to determine the activity of ACCO in the selected ripening stages. A mass of 3 g of freeze-dried fruits and 5 mL of 0.1 M Tris-HCl buffer, pH=8.0 (JT Baker) were placed in 15-mL vials. The reaction vessel was then sealed with a rubber septum and kept in ice; 100 µL of a cold mixture of 5% NaOCl (Sigma-Aldrich, Merck) and saturated NaOH (2:1 *V*/*V*; J.T.Baker) were injected through the septum with a 1-mL syringe. The reaction mixture was shaken on an orbital shaker (MaxQ 4450; Thermo Scientific, Waltham, MA, USA) for 2.5 min, after which 0.5 mL of headspace were injected to an Agilent CG 7820A gas chromatograph (Shanghai, PR China) for ethylene determination using a standard curve (*22*).

### Real time PCR

Total RNA was extracted from transgenic and wild type tomatoes at break stage. Two grams of frozen fruit samples were extracted using the Trizol^®^ reagent (Invitrogen, Carlsbad, CA, USA). The genomic DNA present in the extract was removed by digestion with RNase-free DNase (Invitrogen). Total RNA was quantified and its purity was checked using a Nanodrop 2000^®^ spectrophotometer (Thermo Scientific, Wilmington, DE, USA). One hundred ng of total RNA were used for each 20 µL of reaction volume according to the specifications of PowerSybr Green RNA-to-C_t_ 1-step kit (Applied Biosystems, Vilnius, Lithuania). The reaction mixture consisted of 25 µL of reagent (2×), 1 µL of each primer (10 µM), 0.125 µL RT enzyme mix and nuclease-free water to complete the reaction volume. The reaction protocol entailed a reverse transcription step at 48 °C for 30 min and PCR: initial denaturation step at 95 °C for 10 min and 40 cycles of denaturation and alignment/extension (95 °C for 15 s and 60 °C for 1 min, respectively). Reactions were performed in a StepOne Plus^®^ thermal cycler (Applied Biosystems, Singapore). Oligonucleotides for differentially expressed genes: *ACCO* and *TomloxB* and reference gene: elongation factor 1 (EF1) were synthesized at the Biotechnology Institute of the UNAM in Cuernavaca, Mexico. Sequences were as follows: a) *TomloxB* direct primer: 5’-ATA CAC ACG CGG TGA TCG AA and reverse primer: 5’-AGT TTA GGC CAC CAA GCC TC; b) *ACCO*: direct primer: 5’-TAA TGG GAA TGG GAA GAA AAG ATT and reverse primer: 5’-ACA AAG CAA GAT AAA GCA CCC; and elongation factor: direct primer: 5’-TTC CCT CTA TGC CAG TGG AC and reverse primer: 5’-CAT CTC CAG AG TCC AGC ACA. Data analysis was performed by the StepOne software using the C_t_ method (*23*). A relative quantitation (RQ) value was obtained using the following equation:
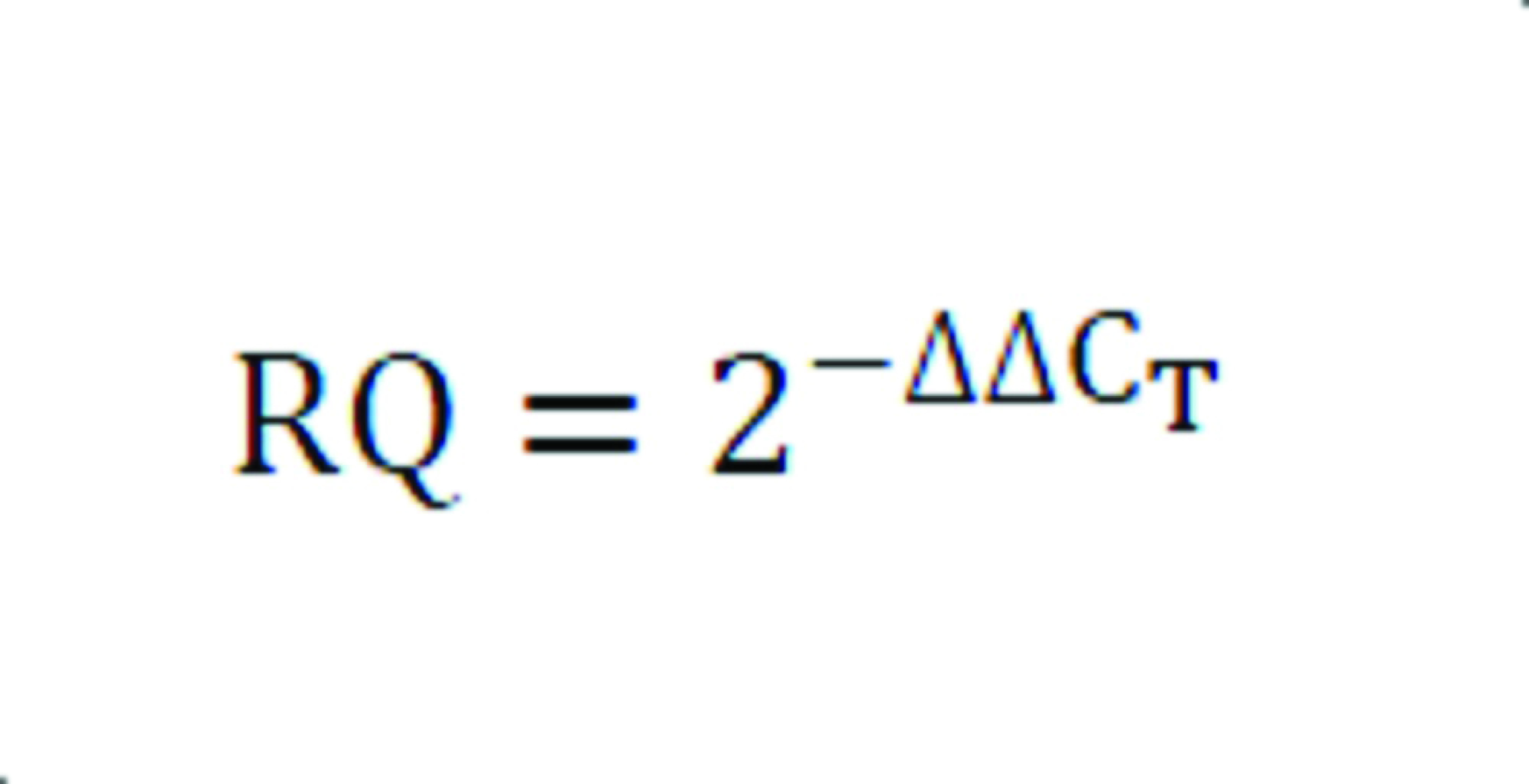
where C_T_ is cycle threshold value.

### Exogenous ethylene application

Experiments were performed at 25 °C using chambers of 0.5 L capacity, to which certified ethylene (Praxair, Azcapotzalco, Mexico City, Mexico) was applied (100 μL/L critical volume fraction in fruits), until a complete change of the internal atmosphere was achieved; 10 min later, ethylene volume fraction was measured until the right value of 100 μL/L was achieved. Tomato fruits were placed inside the chambers and held for 130 min. Genetically modified and wild type tomato fruits exposed to exogenous ethylene were analyzed as described above.

### Statistical analysis

The data were analyzed statistically using the MINITAB 17 software (*24*) to determine the average values and standard errors. One-way ANOVA with the Tukey’s test at a significance level of p=0.05% was performed to assess the difference between treatments.

## RESULTS AND DISCUSSION

### LOX activity as a result of silencing process and after ethylene application

**Fig. 1:**
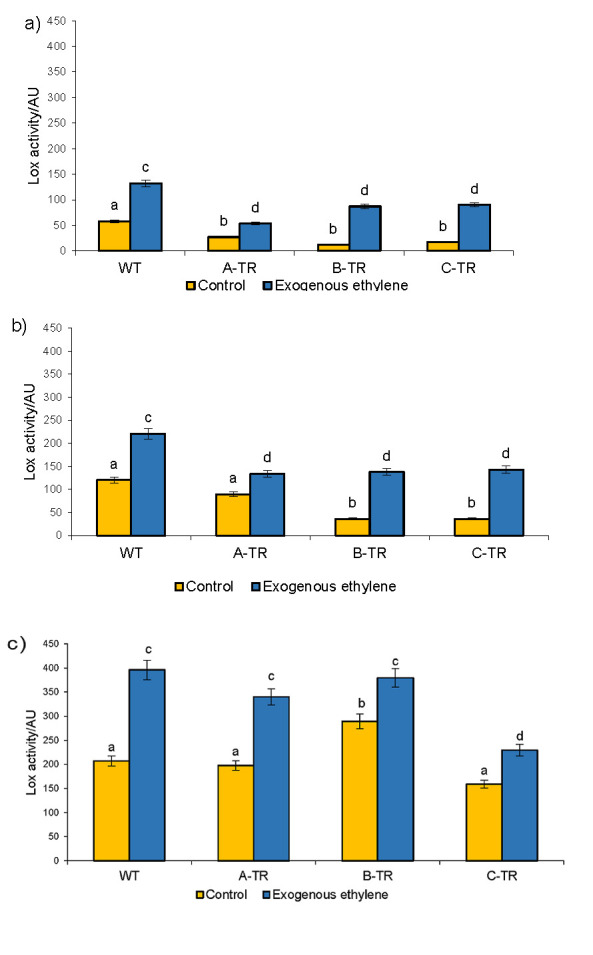
Lipoxygenase (LOX) activity in wild type (WT) and three transgenic lines (A-TR, B-TR and C-TR) in three stages of ripening: a) break, b) turning and c) pink. Fruits without exposure to exogenous ethylene were designated as controls. AU=enzyme activity units. Different letters indicate statistically significant differences (ANOVA, p<0.05)

LOX activity was determined in three ripening stages: break, turning and pink (Fig. 1). In general, the highest activity was found in the pink stage, as reported by several authors (*17*, *18*). However, other authors found the highest activity at the red stage (*1*, *8*). The experiment conditions defined whether LOX activity was carried out immediately after harvest or if fruits were harvested mature and further ripened until they reached the red stage. In the break and turning stages, the transgenic lines (A-TR, B-TR and C-TR) showed a decreased activity compared to the wild type (WT). In contrast, transgenic lines were similar to the WT in the pink stage, except for the B-TR line, which showed a greater activity, as shown in Fig. 1c. The decreased LOX activity in the transgenic lines is very likely a direct consequence of the *TomloxB* gene silencing, as León-García *et al*. (*17*) proved.

The LOX metabolic pathway is triggered when the cell membrane breaks down and hydrolyses the fatty acids of the membrane phospholipids by the action of acyl-hydrolases. Thus, free linoleic and linolenic acids are readily oxidized by the action of lipoxygenase to produce their corresponding 9- or 13-hydroperoxide derivatives, which then serve as substrates for the hydroperoxide lyase pathway (*19*). Therefore, changes in the activity of LOX can determine largely the post-harvest life of the fruit since this enzyme is required for tissue softening and senescence onset.

Application of exogenous ethylene increased the LOX activity in all ripening stages (break, turning and pink), even in the transgenic lines when the gene encoding this enzyme is silenced.

In the break and turning stages, WT had higher values than genetically-modified fruits. These results suggest that ethylene exerts a positive control of the LOX activity. This control seems to be regulated depending on the ripening stage of the fruit. In the pink stage, where the highest activity of the enzyme was found, ethylene exerted a similar effect to that in WT.

### ACC oxidase activity and ethylene production

**Fig. 2:**
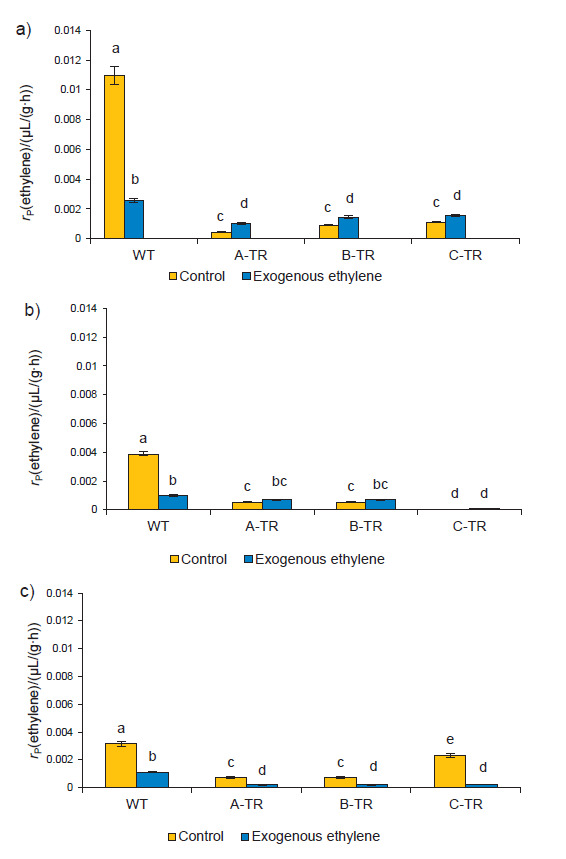
1-Aminocyclopropane-1carboxylic acid (ACC) oxidase activity determined in wild type (WT) and three transgenic lines (A-TR, B-TR and C-TR) in three stages of ripening: a) break, b) turning and c) pink. Fruits without exposure to exogenous ethylene were designated as control. Different letters indicate statistically significant differences (ANOVA, p<0.05)

The highest ACCO activity in both, the transgenic and WT fruits, was obtained in the break stage (Fig. 2a). Biochemical changes in the break stage, driven mainly by ethylene, lead to the fruit ripening. A decrease in the ACCO activity reduces ethylene synthesis (*19*). The application of exogenous ethylene reduced the activity of the ACCO in WT fruits in all three stages (Fig. 2). When the ethylene receptors detect an increase in the concentration of ethylene, the enzyme reduces its activity to slow down the production of the hormone. This is in contrast to what was found in the transgenic lines, where ACCO activity increased only in the break stage. ACCO activity in WT tomatoes and transgenic lines (A-TR, B-TR, C-TR) decreased as ripening progressed (Fig. 2). This corresponds to the maximum production of ethylene in the break stage, which is discussed below, since it is the ACCO that regulates the last step in the production of ethylene. However, when exogenous ethylene was applied in transgenic fruits, an increase in the ACCO activity was observed in the break stage (Fig. 2a) because the application of ethylene affects the ripening of climacteric fruits. This occurs by the regulation of gene expression which results in transcript expression and protein synthesis necessary to accelerate maturation. However, for this to happen there must be a minimum concentration of ethylene for the receptors to trigger the necessary reactions in order to increase maturation. Research shows that 100 µL/L of ethylene is the minimum for the receptors to trigger the synthesis of the enzymes necessary to accelerate maturation (*25*).

In our study, ACCO activity was reduced as a consequence of silencing of the *TomloxB* gene, because the silencing mode reduces the number of transcripts and the synthesis of lipoxygenases, which catalyze the peroxidation of polyunsaturated fatty acids (linoleic and linolenic acid) found in the membrane of plant cells (*26*). The main products of the oxidation of these fatty acids are free radicals that contain reactive oxygen species, called free radicals, mainly hydroxyl (˙OH), superoxide (O_2_^∙^ˉ) and hydrogen peroxide (H_2_O_2_) (*27*), which, in addition to affecting the stability of the cell membrane, also upset the function of certain oxygen-dependent enzymes such as ACCO, which in turn, participate in the Yang cycle, producing ethylene from methionine (*22*). At low ethylene synthesis, the shelf life of the fruit can increase. In this case, the silencing mode of *TomloxB* affects the activity of the ACCO with the concomitant reduction of ethylene. These results may indicate that ethylene regulates the concentration of lipoxygenase in the break stage, as reported by Griffiths *et al*. (*1*).

**Fig. 3:**
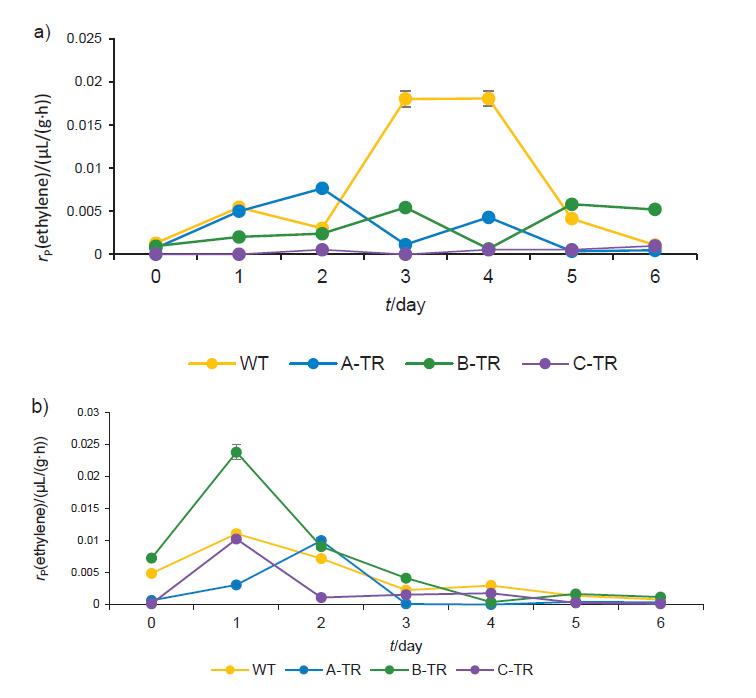
Ethylene production in the break stage of ripening of wild type and three lines of transgenic fruits (A-TR, B-TR and C-TR): a) untreated fruits, and b) fruits treated with exogenous ethylene.

Ethylene production was measured in the break stage (Fig. 3). In the treatments not including the application of exogenous ethylene (Fig. 3a) a reduction in the production of ethylene can be seen in the genetically modified fruits (A-TR, B-TR and C-TR). In this case, the climacteric peak, which consists of the drastic increase in the production of ethylene at the beginning of the ripening (*28*), was observed between the third and fourth day. However, in the fruits treated with exogenous ethylene, as shown in Fig 3b, the concentration of ethylene in WT fruits decreased, whereas it increased in the genetically modified fruits. These results are consistent with the profile of ACCO activity. The lower the ethylene synthesis, the longer the expected shelf life of the fruit. In this case, silencing of the *TomloxB* gene decreased not only the LOX activity, but also the ethylene production and ACCO activity. Ethylene can promote its own production by autocatalysis, which is, in some cases, independent of the applied exogenous ethylene concentration (*29*), or prevent its synthesis by self-inhibition (*29*, *30*). This will depend on the concentration of exogenous ethylene and the time of exposure (*31*). It is possible that a minimum content (100 µL/L) exists at which ethylene can promote its own synthesis; hence, the transgenic lines showed an increase in ethylene attributed to its low content in the LOX-silenced fruits (*32*, *33*).

Our results agree with those of Griffiths *et al*. (*1*), who found that TomloxB activity increased after the addition of ethylene. It is possible that ethylene modulated TomloxB depending on the ripening stage, as shown in Fig. 1a. Likewise, TomloxB affects ethylene production, because silencing *TomloxB* gene reduced the climacteric peak height, as shown in Fig. 3b.

**Fig. 4:**
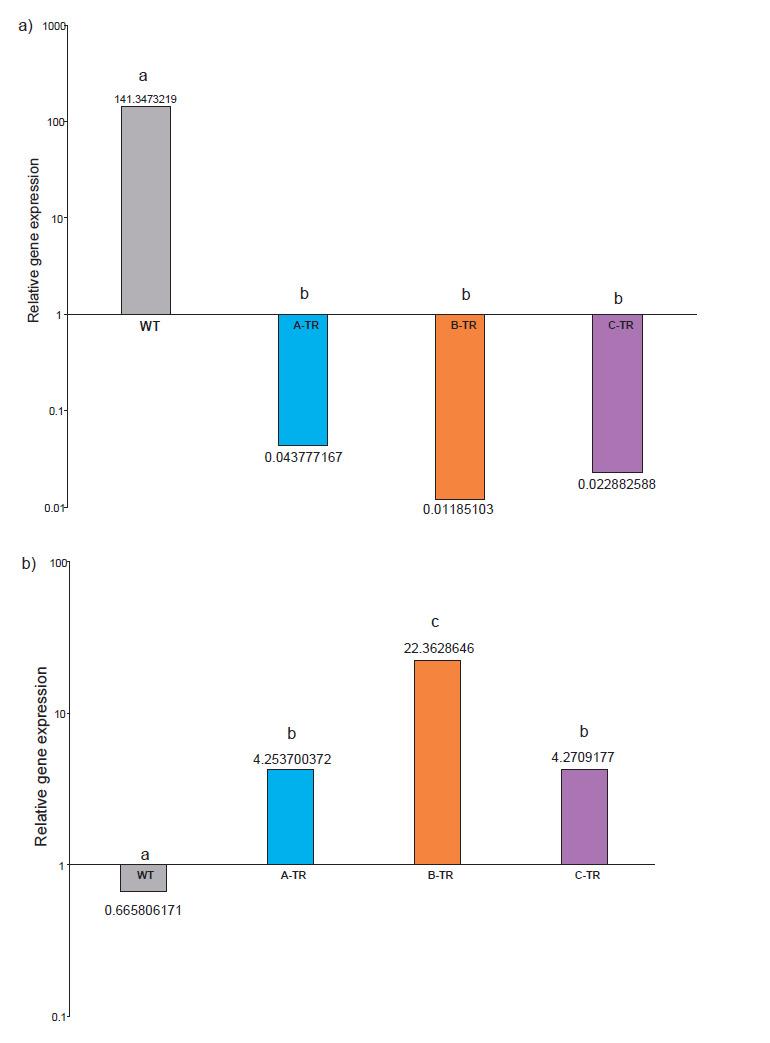
Differences in gene expression of: a) *ACCO* and b) *TomloxB* genes obtained by substracting the gene expression without exogenous ethylene from the gene expression with exogenous ethylene for each strain. Different letters indicate statistically significant differences (ANOVA, p<0.05)

Relative gene expression for *ACCO* and *TomLoxB* (Fig. 4) was calculated by subtracting normalized cycle threshold (C_T_) values of fruits without exogenous ethylene treatment to normalized C_t_ values of fruits with exogenous ethylene treatment.

*ACCO* gene expression diminished in WT tomatoes treated with ethylene (due to the inhibitory effect that ethylene exerts on the tissue), but in the transgenic lines this expression was higher in fruits exposed to ethylene than in the fruits without ethylene treatment (Fig. 4a) and it coincides with the activity of the ACCO enzyme determined in the break stage shown in Fig. 2a, suggesting that ethylene had an effect on the ACCO regulatory system, as it has been previously discussed (*19*, *34*). These fruits had a reduction in ethylene production due to the silencing of the *TomloxB*. On the other hand, as shown in Fig. 4b, *TomloxB* expression in WT increases in fruits without ethylene, while the expression in transgenic lines was reduced in fruits treated with ethylene, thus confirming the effect of gene silencing and suggesting that exogenous ethylene had no effect on these genetically modified fruits like on wild type fruits in break stage. The effect of the application of ethylene can probably be seen in the expression of the *TomloxB* gene in the pink stage, as shown in Fig. 1c, where maximum enzyme activity was found in ethylene-treated fruits. Nevertheless, it is important to take into consideration that even though the *TomloxB* expression was reduced in transgenic lines, the overall LOX activity increased with the application of ethylene. This may be because TomloxB is not the only LOX enzyme present in the fruit and while its encoding gene was silenced, its individual enzymatic activity was reduced. This may be not true for the other LOX present in the fruit, which remained active and sensitive to ethylene. This finding points out to the specificity of the silencing of the described *TomloxB* (*17*); however, further work should be pursued to confirm it. Another explanation could be that the technique for the determination of the LOX enzyme is not specific to TomloxB and the result of the activity could reflect the activity of more than one isoform. However, the isoform that has been reported in the fruit is TomloxB (*1*, *7*, *35*). These two datasets, as well as the data observed and discussed previously in enzymatic activity support the idea that silencing *TomloxB* does not impair the ability of fruit to respond to exogenous ethylene in break stage. This could mean that *TomloxB* makes transcripts for lipoxygenase production, and it can regulate the production of ethylene. A reduction in LOX activity reduced ethylene production in break stage, while the addition of exogenous ethylene increased LOX activity in break, turning and even more in pink stage. Therefore, this regulation depends on the stage of ripening, ethylene concentration and exposure time. The results suggest that lipoxygenase may be a regulator of ACCO and production of ethylene in break stage. These results are promising because they could lead to a better understanding of the metabolic contribution of TomloxB in fruit ripening and how it is linked to senescence-related process, which can lead to longer shelf life of fruits.

## CONCLUSION

Both TomloxB and ethylene have an essential function in the ripening process. *TomloxB* and *ACCO* genes are expressed differently during fruit ripening, but the break stage may be an important regulation phase between LOX and ethylene. Decreasing LOX activity is an alternative to delaying the ripening of climacteric fruits such as tomatoes, reducing ACCO activity and also ethylene production. These results provide a basic understanding of the fruit quality as related to LOX activity and ethylene.
